# Functional Analysis of Conserved Motifs in Influenza Virus PB1 Protein

**DOI:** 10.1371/journal.pone.0036113

**Published:** 2012-05-15

**Authors:** Caroline Chu, Shufang Fan, Chengjun Li, Catherine Macken, Jin Hyun Kim, Masato Hatta, Gabriele Neumann, Yoshihiro Kawaoka

**Affiliations:** 1 Department of Pathobiological Sciences, School of Veterinary Medicine, Influenza Research Institute, University of Wisconsin-Madison, Madison, Wisconsin, United States of America; 2 Theoretical Biology and Biophysics, Los Alamos National Laboratory, Los Alamos, New Mexico, United States of America; 3 Division of Virology, Department of Microbiology and Immunology, Institute of Medical Science, University of Tokyo, Tokyo, Japan; University of Hong Kong, Hong Kong

## Abstract

The influenza virus RNA polymerase complex is a heterotrimer composed of the PB1, PB2, and PA subunits. PB1, the catalytic core and structural backbone of the polymerase, possesses four highly conserved amino acid motifs that are present among all viral RNA-dependent RNA polymerases. A previous study demonstrated the importance of several of these conserved amino acids in PB1 for influenza polymerase activity through mutational analysis. However, a small number of viruses isolated in nature possesses non-consensus amino acids in one of the four motifs, most of which have not been tested for their replicative ability. Here, we assessed the transcription/replication activities of 25 selected PB1 mutations found in natural isolates by using minireplicon assays in human and avian cells. Most of the mutations tested significantly reduced polymerase activity. One exception was mutation K480R, observed in several pandemic (H1N1) 2009 viruses, which slightly increased polymerase activity relative to wild-type. However, in the background of the pandemic A/California/04/2009 (H1N1) virus, this mutation did not affect virus titers in cell culture. Our results further demonstrate the functional importance of the four conserved PB1 motifs in influenza virus transcription/replication. The finding of natural isolates with non-consensus PB1 motifs that are nonfunctional in minireplicon assays suggests compensatory mutations and/or mixed infections which may have ‘rescued’ the inactive PB1 protein.

## Introduction

Influenza A viruses possess an RNA genome consisting of eight single-stranded gene segments, each encoding at least one viral protein [1]. In the virus particle, individual segments are assembled into ribonucleoprotein (RNP) complexes that contain viral RNA (vRNA) encapsidated by nucleoprotein (NP) and an associated RNA-dependent RNA polymerase. As a heterocomplex, the polymerase consists of three virus encoded polypeptides: polymerase basic protein 1 (PB1), polymerase basic protein 2 (PB2), and polymerase acidic protein (PA). Upon infection, the viral RNPs migrate into the host cell nucleus where negative-sense vRNA serves as a template for synthesis of positive-sense messenger RNA (mRNA) and complementary RNA (cRNA) through the mechanisms of transcription and replication, respectively [2]. Initiation of viral transcription requires 5′-cap primers generated from host cell pre-mRNAs [3]. Cap binding and cleavage activities are performed by the viral polymerase complex and depend on the interaction of the complex with the vRNA template [4]. Polyadenylation of the viral mRNA transcripts occurs by reiterative copying of an oligo(U) sequence adjacent to the 5′ terminus of each vRNA [5], [6]. In addition, the viral polymerase complex generates full-length, uncapped copies of cRNA, which act as the replicative intermediate for production of progeny vRNAs that are assembled into new virions [7].

While all three polymerase proteins are required for efficient replication and transcription in virus-infected cells [8], PB1 plays a central role in the formation of the structural backbone and catalytic activities of the RNA polymerase [2], [9]. It possesses four highly conserved regions of amino acids (motifs I, II, III, and IV, ranging in size from 4–13 amino acids) identified by comparative sequence analysis of all viral RNA-dependent DNA polymerases and RNA-dependent RNA polymerases [10], [11]. Together, these four conserved motifs form a large functional domain with at least one ‘invariant’ amino acid per motif [11], [12], [13]. In a previous study [10], these ‘invariant’ amino acids were tested for their significance in polymerase activity in a minireplicon assay; additional mutations were also introduced into the influenza viral PB1 protein to resemble amino acid sequences found in the polymerases of other RNA viruses. Most of these mutations significantly reduced polymerase activity, demonstrating the critical role of the conserved motifs in PB1 for influenza virus transcription and replication [10]. However, our inspection of influenza A virus PB1 sequences revealed a small number of PB1 proteins with non-consensus amino acids in the conserved motifs. Currently, it remains unknown whether these PB1 proteins support efficient viral replication and transcription of influenza viral RNAs. Here, we therefore tested selected PB1 variants for their replicative ability in minireplicon assays in cell culture. Our data show that these amino acid changes may significantly inactivate the polymerase, providing further support for the importance of these four conserved PB1 motifs for influenza polymerase activity.

## Materials and Methods

### Cell Culture

293T human embryonic kidney cells (obtained from ATCC) and DF-1 chicken fibroblast cells (obtained from ATCC) were maintained in Dulbecco’s modified Eagle’s medium (DMEM) supplemented with 10% fetal bovine serum (FBS). Calu-3 human airway epithelial cells (obtained from Raymond Pickles, University of North Carolina, Chapel Hill, NC) and Madin-Darby canine kidney (MDCK, obtained from ATCC) cells were grown in DMEM/F-12 [Nutrient Mixture F-12] supplemented with 10% FBS and in minimal essential medium (MEM) containing 5% newborn calf serum, respectively. Unless otherwise indicated, all cell cultures were incubated at 37°C in 5% CO_2_.

### Plasmid Construction

The construction of eukaryotic protein expression plasmids pCAGGS/MCS [14] encoding for virus polymerase (PB2, PB1, and PA) and NP proteins of A/bar-headed goose/Qinghai/1/2005 (H5N1) was performed as previously described [15]. In brief, full length cDNA of each polymerase and NP gene was generated from extracted viral RNA (vRNA) by RT-PCR. Protein expression plasmids were then constructed by cloning the entire cDNA into the pCAGGS/MCS vector controlled by the chicken β-actin promoter. PB1 mutant plasmids were generated by site-directed mutagenesis PCR using primers containing the corresponding alterations. All plasmid constructs were subsequently sequenced to confirm the presence of the mutation of interest, and the absence of unwanted mutations.

To assess the replicative ability of the viral polymerase complex in human or avian cells, cDNA constructs transcribing virus-like RNA that encodes the reporter protein luciferase were cloned under the control of the human [16] or avian [17] RNA polymerase I promoter, respectively.

For generation of recombinant influenza viruses, genes of the A/California/04/2009 (H1N1) virus were cloned into an RNA polymerase I (pol I)-driven plasmid, as described previously [16]. Briefly, cDNAs for the full-length viral RNAs of each gene were amplified by PCR with primers containing *Bsm*BI sites, incubated with *Bsm*BI, and cloned into the *Bsm*BI-sites of pPol I vectors. In these RNA polymerase I constructs, each gene is consequently flanked by the human RNA pol I promoter and the mouse RNA pol I terminator. All constructs were sequenced prior to use to confirm the desired sequence.

### Expression and Measurement of Luciferase Activity

Human 293T cells in 24-well culture plates were transfected with virus polymerase protein expression plasmids (i.e., PB2, PA, NP, and wild-type or mutant PB1, 0.1 µg each), along with the luciferase reporter plasmid (0.02 µg) controlled by the human RNA pol I promoter. As an internal control for the luciferase assay, *Renilla* luciferase reporter plasmid pGL4.74[hRluc/TK] (0.01 µg) (Promega, Madison, WI) was also cotransfected into cells to normalize for transfection efficiency. At 24 h post-transfection, cells were lysed and assessed for luciferase activity using the Dual-Luciferase Reporter Assay System (Promega) and automated application of the Infinite M1000 microplate reader (Tecan, Switzerland) according to manufacturer’s instructions. Three independent experiments were carried out, each performed in triplicate. Luciferase activity levels were expressed as percentages of the values observed for wild-type PB1.

Assays in avian cells were essentially carried out as described above. Chicken DF-1 cells were transfected with the four protein expression plasmids (0.25 µg each), the luciferase reporter plasmid controlled by the avian RNA pol I promoter (0.05 µg), and pGL4.74[hRluc/TK] (0.025 µg). DF-1 cells were incubated at 41°C for 24 h to accommodate for the higher body temperature of chickens. Luciferase activities were determined as described above.

### Quantitative Real-time RT-PCR

Human 293T cells in 6-well culture plates were transfected with polymerase and NP protein expression plasmids (i.e., BHG/01-pCAGGS-PB2, BHG/01-pCAGGS-PA, BHG/01-pCAGGS-NP, and wild-type or mutant BHG/01-pCAGGS-PB1, 2 µg each), together with an RNA transcription plasmid (polI-WSN-NP, 0.5 µg) controlled by the human RNA pol I promoter. Eight hours later, cells were washed three times and incubated with Opti-MEM medium. Forty-eight hours after transfection, cells were harvested and lysed by direct addition of RLT buffer from the RNeasy kit (QIAGEN), followed by RNA extraction and DNase treatment to remove transfected plasmid DNA. The concentration of purified RNA was determined by use of spectrophotometry.

Hot-start reverse transcription with specific primers for the amplification of vRNA, cRNA, and mRNA was performed as described by Kawakami *et al*. [18]. Quantitative PCR (qPCR) was performed with SYBR Green qPCR SuperMix for ABI PRISM (Invitrogen) on an ABI PRISM 7900HT cycler. For qPCR amplification, samples were incubated for 10 min at 95°C, followed by 40 cycles of 95°C for 15 s and 60°C for 1 min. The amount of beta-actin mRNA served as internal control. The relative concentrations of viral replication products were determined by analysis of cycle threshold values, normalized to beta-actin, and then calculated as percentages relative to the levels of wild-type RNAs. Results represent the average levels of RNAs ± standard deviations from one representative experiment carried out in triplicate.

### Western Blot Analysis

293T cells in 6-well culture plates were transfected with plasmids expressing wild-type or mutant PB1 protein. Forty-eight hours later, cells were collected, pelleted by centrifugation at 3,000 rpm for 5 min, lysed in RIPA buffer [25 mM Tris-HCl pH 7.6, 150 mM NaCl, 1% NP-40, 1% sodium deoxycholate, 0.1% SDS, protease inhibitor cocktail tablets (Roche, Indianapolis, IN)] for 20 min on ice, and spun down at 4°C for 15 min. Supernatants were mixed with SDS loading buffer (160 mM Tris-HCl, pH 6.8, 4% SDS, 20% glycerol, 10% 2-mercaptoethanol, and 0.04% bromophenol blue) and blotted. PB1 protein and beta-actin were detected with antibodies to the respective proteins (available in our group or purchased from Sigma, St. Louis, MO, respectively).

### Generation of Viruses by Plasmid-based Reverse Genetics

Wild-type and mutant A/California/04/2009 (H1N1) viruses were generated as previously described [16], [19]. Briefly, 293T cells were transiently transfected with eight RNA polymerase I plasmids (0.1 µg each) expressing each of the influenza vRNA segments and four pCAGGS/MCS plasmids (1 µg each) designed to express the viral PB2, PB1, PA, and NP proteins. Viral supernatants were harvested at 48 h post-transfection and amplified in MDCK cells to produce viral stocks. The PB1 genes of stock viruses were sequenced to rule out unwanted mutations. The titers of virus stocks were determined by plaque assays in MDCK cells.

### Replicative Properties of Viruses

Confluent monolayers of human Calu-3 cells were infected with a multiplicity of infection (MOI) of 0.001 of virus. After 1 h incubation, the cells were washed and further incubated in MEM/F12 medium containing 0.3% bovine serum albumin and TPCK-treated trypsin (0.4 µg/ml) at 35°C. Aliquots of supernatants were harvested for virus titration every 12 h over an 84 h time course post-infection: 12 h, 24 h, 36 h, 48 h, 64 h, 72 h, and 84 h. Virus titers for each time point were determined by plaque assays in MDCK cells.

## Results

### Influenza A Virus PB1 Proteins Possessing Non-consensus Amino Acids in the Four Conserved Polymerase Motifs

**Figure 1:**
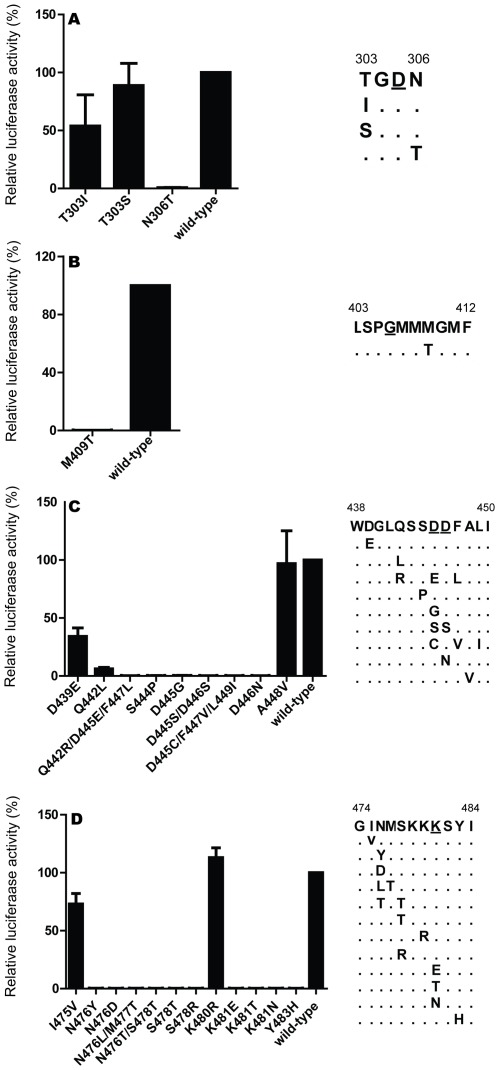
Effects of mutations in the four conserved motifs of PB1 on influenza polymerase activity. (**A**) Motif I; (**B**) Motif II; (**C**) Motif III; (**D**) Motif IV. Consensus sequences are listed by motif with numbers at each end indicating the position of the first and last amino acid residue. Invariant amino acids are underlined. Selected mutations found in the four conserved motifs of natural isolates (see **Table 3**) were introduced into the PB1 protein of A/bar-headed goose/Qinghai/1/2005 (H5N1) virus. The polymerase activity of each PB1 mutant was assessed in human 293T cells by quantifying luciferase activity in the cell lysates of cells transfected with PB1- (wild type or mutant), PB2-, PA- and NP-protein expression plasmids along with a reporter plasmid expressing an influenza virus-like RNA construct for luciferase. Luciferase activity levels were normalized by the *Renilla* internal control and expressed as a percentage relative to wild-type PB1 activity set as 100%. Data values are averaged over three independent transfection experiments where each experiment was run in triplicate. Error bars represent one standard deviation.

Based on our analysis and information published by Biswas & Nayak [10], we evaluated the following amino acid motifs in the influenza virus polymerase protein PB1: motif I –^303^TGDN^306^; motif II –^403^LSPGMMMGMF^412^; motif III –^438^WDGLQSSDDFALI^450^; motif IV –^474^GINMSKKKSYI^484^ (see also **Fig. 1A–D**); these motifs are highly conserved among influenza A virus PB1 proteins, while minor deviations exist for the PB1 proteins of influenza B and C viruses. We inspected the Los Alamos National Laboratory influenza database (http://www.flu.lanl.gov/; sequences used in this research are available to the public and can be requested by emailing flu@lanl.gov”) and the NIH NCBI (http://www.ncbi.nlm.nih.gov/genomes/FLU/FLU.html) influenza database and identified ∼235 mutations in the conserved motifs of influenza A virus PB1 proteins (among >14,000 PB1 proteins listed) (**Table 1**). The most frequently observed deviation was K480R (motif IV), which was found in 33 human, 40 swine, and 15 avian virus PB1 proteins (i.e., in 0.45%, 4.21%, and 0.24% of the evaluated human, swine, and avian virus PB1 proteins, respectively).

**Table 1:** Influenza A virus PB1 proteins with mutations in the conserve motifs I – IV.

Originof PB1	Sequences evaluated	PB1 proteins with mutations in				
		motif I	motif II	motif III	motif IV	Total
Human virus	7218 (49.6%*)	4	3	8	39	54 (23%*)
Avian virus	6188 (42.5%*)	8	19	36	57	120 (51%*)
Swine virus	950 (6.5%*)	2	3	3	48	56 (24%*)
Other origin	208 (1.4%*)	0	4	1	0	5 (2%*)
Total	14564 (100%)	14	29	48	144	235 (100%)

* Percentage of total changes.

Comparison of the four conserved motifs revealed lower variability in motifs I and II (14 and 29 mutations, respectively) than in motif III (48 mutations) and motif IV (144 mutations, of which 88 encode K480R, see above) (**Table 1**). Avian virus PB1 proteins show greater variability in the conserved motifs than human virus PB1 proteins (**Table 1**), which can be traced to a relatively high variability in the conserved PB1 domains of viruses of the H5N1 and H9N2 subtypes (**Table 2**): for example, 50 of the 120 mutations (i.e. 42%) found in PB1 motifs I – IV map to H5N1 viruses, although viruses of this subtype account for only 25% of the avian virus sequences in the database. Among human influenza viruses, greater variability in PB1 motifs I-IV was found among pandemic (H1N1) 2009 viruses as compared to seasonal H1N1 or H3N2 viruses.

**Table 2:** Avian virus PB1 proteins with mutations in the conserved motifs I – IV.

Virussubtype	Sequences evaluated	PB1 proteins with mutations in				
		motif I	motif II	motif III	motif IV	Total
H5N1	1562 (25%*)	5	2	15	28	50 (42%*)
H9N2	774 (13%*)	0	4	9	15	28 (23%*)
H7Nx	626 (10%*)	1	1	1	1	4 (3%*)
H6Nx	663 (11%*)	1	1	1	5	8 (7%*)
All Other	2563 (41%*)	1	11	10	8	30 (25%*)
Total	6188 (100%)	8	19	36	57	120 (100%)

* Percentage of total changes.

Past analyses also revealed one or two ‘invariant’ amino acids per motif [10]. We identified two, one, seven, and seven PB1 proteins that do not encode the ‘invariant’ residues D305 (motif I), G406 (motif II), D445/D446 (motif III), or K481 (motif IV), respectively. These mutations were primarily found in avian virus PB1 proteins (14 mutations), in contrast to only three and no mutations in swine and human virus PB1 proteins, respectively.

Given the high degree of conservation for PB1 motifs I – IV, multiple changes may be considered unlikely. Nonetheless, we identified 22 PB1 proteins that possess more than one alteration in one of the conserved motifs, and 10 PB1 proteins that possess alterations in two motifs. Again, most of these proteins (19/22, and 7/10, respectively) are of avian virus origin.

Collectively, our analyses indicated that influenza A viruses with alterations in the conserved polymerase motifs of PB1 are found in nature.

### Polymerase Complex Activity of Wild-type and Mutant PB1 Proteins in Human Cells

To assess the replicative ability of PB1 proteins possessing non-consensus amino acids, we selected 25 variants (**Table 3**) for further testing. We focused on variants that occurred in more than one virus isolate, highly pathogenic avian H5N1 viruses, at the ‘invariant’ positions in motifs III or IV (viruses with deviations at the ‘invariant’ positions in motif I or II had not been reported at the time variants were selected for testing), and/or were detected in the PB1 proteins of viruses that crossed the species barrier, such as (*i*) A/Guangxi/1/05, A/Indonesia/CDC292N/05, and A/Indonesia/286H/06 (avian H5N1 influenza viruses isolated from infected individuals), (*ii*) A/Thailand/271/05 (H1N1; a swine influenza virus isolated from an infected person), and (*iii*) A/swine/Anhui/ca/2004 (H5N1) and A/swine/Korea/S83/2004 (H9N2), both of which are avian influenza viruses that were transmitted to pigs.

**Table 3:** Mutations in the conserved motifs I – IV of the PB1 protein and their effects on replicative ability in human and chicken cells.

Motif	Mutation(s)	Mutant BHG polymerase activity (%)^1^		Virus(es) possessing the respective mutation(s)
		Human cells	Chicken cells	
	T303I	27.2	53.9	A/chicken/Taiwan/G2/87 (H6N1)A/duck/HK/412/78 (H4N2)
I	T303S	78.9	88.8	A/Guangxi/1/05 (H5N1)
	N306T	2.4	0.8	A/Thailand/271/05 (H1N1)
II	M409T	0	0.2	A/swine/Nordkirchen/IDT1993/03 (H3N2)
	D439E	49.0	34.2	A/bar-headed goose/Qinghai/1/05 (H5N1)A/whooper swan/Qinghai/01/05 (H5N1)
	Q442L	19.2	6.2	A/duck/Vietnam/8/05 (H5N1)A/duck/Vietnam/GZ-8/05 (H5N1)A/Hong Kong/483/97 (H5N1)
	Q442R/D445E/F447L	0	0.3	A/duck/Hubei/wq/03 (H5N1)
	S444P	0	0.4	A/chicken/Anhui/1089/07 (H5N1)
III	D445G	0	0.5	A/mallard/Yan chen/05 (H4N6)
	D445S/D446S	0	0.3	A/swine/Doetlingen/IDT4735/2005 (H1N2)
	D445C/F447V/L449I	0	0.3	A/chicken/Shantou/1126/01 (H9N2)
	D446N	0	0.3	A/chicken/Guangdong/5/97 (H9N2)A/chicken/Guangdong/10/00 (H9N2)A/chicken/Jiangsu/1/00 (H9N2)
	A448V	40.8	96.9	A/swine/Shanghai/1/07 (H1N2)A/swine/North Carolina/93523/01 (H1N2)
	I475V	67.6	73.1	A/Indonesia/CDC292N/05 (H5N1)A/Queensland/60/05 (H3N2)A/chicken/Taiwan/2838N/00 (H6N1)
	N476Y	0	0.3	A/swine/Anhui/ca/04 (H5N1)
	N476D	0	0.3	A/Indonesia/286H/06 (H5N1)A/chicken/Taiwan/G2/87 (H6N1)
IV	N476L/M477T	0	0.4	A/great black-headed gull/Qinghai/2/05 (H5N1)
	N476T/S478T	0	0.3	Four 2005 H5N1 (Qinghai-Lake) isolates^2^
	S478T	0	0.3	Four 2005 H5N1 (Qinghai-Lake) isolates^2^
	S478R	0	0.2	A/swine/Guangxi/wz/04 (H5N1)
	K480R	117.2	113.1	Several pandemic (2009) H1N1 viruses; several swine viruses
	K481E	0	0.3	A/duck/Hunan/3340/06 (H5N1)
	K481T	0	0.2	A/chicken/Hong Kong/873.3/01 (H5N1)
	K481N	0	0.3	A/turkey/Israel/1567/04 (H9N2)
	Y483H	0.5	0.3	A/swine/Korea/S83/04 (H9N2)

1 The indicated mutations were introduced into the PB1 protein of A/bar-headed goose/Qinghai/1/2005 (H5N1; BHG) virus. Polymerase activity in human 293T and avian DF-1 cells is indicated as percentage relative to wild-type PB1 activity.

2 For some of these isolates, different sequences are reported in the Los Alamos National Laboratory and NIH NCBI databases.

To determine the transcription/replication efficiency of wild-type or altered polymerase complexes, we introduced the selected changes into the PB1 segment of A/bar-headed goose/Qinghai/1/2005 (H5N1; BHG) virus through site-directed mutagenesis. This strain was chosen because it possesses consensus sequences at all four conserved motifs, and is closely related to some of the viruses in which several of the selected mutations were found [20]. To assess the replicative ability of wild-type and mutant PB1 proteins, we transfected human 293T cells with four plasmids encoding BHG-PB2, -PA, -NP, and wild-type or mutant PB1 protein, and a virus-like RNA encoding the reporter luciferase.

The results obtained in three independent experiments (each carried out in triplicate) are summarized in **Fig. 1A–D** and **Table 3**. The polymerase activities of the BHG PB1 mutants tested can be classified into five categories: (i) seventeen mutants with no or residual activity (luciferase values less than 1% compared to wild-type PB1); (ii) two with drastically reduced activity (between 1 and 20%); (iii) three with moderately reduced activity (between 21 to 60%); (iv) two with slightly reduced or equivalent activity (between 61 to 100%), and (v) one with slightly higher activity than wild type. Thus, most of the mutations tested significantly decreased or completely eliminated the BHG polymerase activity in our assay, despite detection of the particular amino acids in natural isolates. Notably, replacement of the invariant amino acids D445 and D446 in motif III, and K481 in motif IV, abolished polymerase activity entirely. In contrast, mutation K480R (motif IV) slightly increased transcription/replication activity relative to wild-type polymerase activity.

**Figure 2:**
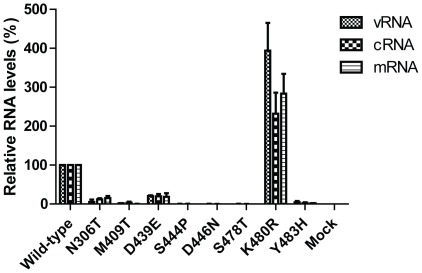
Quantitative analysis of vRNA, cRNA, and mRNA levels for wild-type and mutant PB1 proteins. 293T cells were transfected with plasmids expressing PB2, PA, NP, and wild-type or mutant PB1 protein, along with a plasmid transcribing a viral RNA. The levels of vRNA, cRNA, and mRNA were assessed by quantitative RT-PCR and compared to those obtained with wild-type PB1 protein. The results shown represent the average levels of RNAs ± standard deviations from a representative experiment carried out in triplicate. As a negative control (Mock), the PB1 protein expression plasmid was omitted.

For selected mutants, we also carried out quantitative real-time RT-PCR to assess the levels of vRNA, cRNA, and mRNA in transfected 293T (**Fig. 2**). The K480R mutant yielded increased levels of viral replication products compared to wild-type PB1; this increase was even more pronounced than that detected in luciferase assays. For the other mutants tested, the levels of viral replication products overall correlated with the levels of luciferase expression: the N306T and D439E mutants yielded reduced levels of vRNA, cRNA, and mRNA, whereas the remaining mutants did not catalyze the synthesis of appreciable amounts of viral replication products. Taken together, these results suggested that the conserved motifs in PB1 indeed play a significant role in the replicative capability of the influenza polymerase complex.

**Figure 3:**
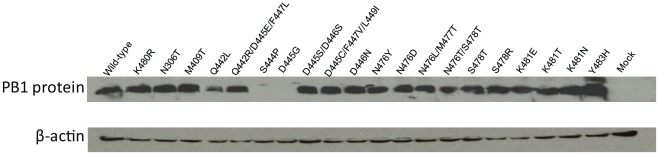
Western blot analysis of wild-type and mutant PB1 proteins. 293T cells were transfected with plasmids expressing wild-type or mutant PB1 protein. Western blot analysis was carried out with antibodies to PB1 and beta-actin (control).

To assess if the impaired replicative ability of some PB1 proteins resulted from reduced protein stability, we next performed western blot analysis for PB1 mutants with significantly reduced replicative ability (**Fig. 3**); the K480R mutant was included as a second ‘positive control’ since it directed efficient genome amplification and would thus be expected to be expressed stably. We found that the S444P and D445G mutations rendered the protein unstable; however, other mutations at position 445 (tested in combination with mutations at position 446, or at positions 447 and 449) resulted in detectable protein, suggesting that the nature of the amino acid at this position is critical. The remaining mutants tested here were detected by western blot analysis, indicating that their polymerase activity (rather than their stability) was affected.

### Polymerase Complex Activity of Wild-type and Mutant PB1s in Avian Cells

In the experiments described above, we measured the transcription/replication activity of wild-type and mutant avian influenza virus polymerase proteins in human cells. Since the replicative ability of influenza viral polymerase complexes can differ significantly between avian and human cells [21], [22], we next carried out minireplicon assays in chicken cells (DF-1) using a virus-like RNA synthesized from an avian RNA polymerase I promoter [17]. Overall, we observed the same trends of polymerase activity (**Table 3**); however, two mutant PB1 proteins (T303I and A448V) showed higher activity in chicken than in human cells, while the inverse effect was observed for the D439E and Q442L mutants. As detected in human 293T cells, mutant K480R once again augmented transcription and replication activity slightly. Taken together, these data argued against host-specific effects for the PB1 mutants tested. Since DF-1 cells were maintained at 41°C following transfection, these results also suggested that these PB1 mutants were active at the body temperature of chickens.

### Polymerase Complex Activity and Virus Replication of PB1-K480R in Pandemic H1N1 Virus Background

**Figure 4:**
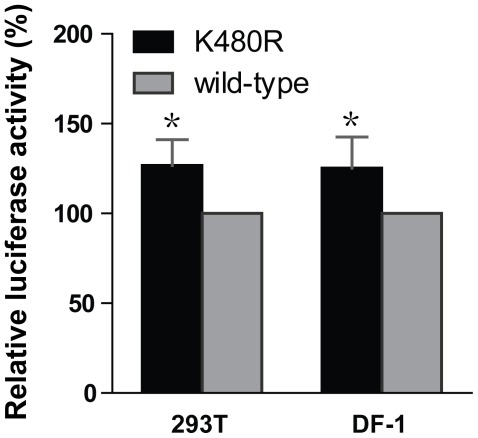
Effects of PB1 mutation K480R on influenza polymerase activity in human and avian cells. The effects of PB1 mutation K480R were evaluated in the background of the A/California/04/09 (H1N1) virus replication complex in human 293T and chicken DF-1 cells. The experiments were carried out as described in the legend to Figure 1. Luciferase activity levels were normalized by the *Renilla* internal control and expressed as a percentage relative to wild-type PB1 activity in the particular cell line. *P<0.07 by Student’s t-test.

Among the 25 PB1 mutants tested, only K480R increased the replicative ability of the BHG polymerase complex in minireplicon assays. Interestingly, this mutation has been observed in several early pandemic (H1N1) 2009 virus isolates. The K480R mutation may have originated in pig influenza viruses, where it has been found more frequently than in human and avian virus PB1 proteins. To assess its effects on the replicative ability of a pandemic (H1N1) 2009 virus, we evaluated this mutation in 293T and DF-1 cells in the background of A/California/04/2009 (H1N1; CA04) virus, an early pandemic (H1N1) 2009 virus which possesses consensus sequences at all four PB1 motifs. As found in the background of BHG, the K480R mutation slightly increased polymerase activities relative to wild-type in both cell lines (**Fig. 4**).

**Figure 5:**
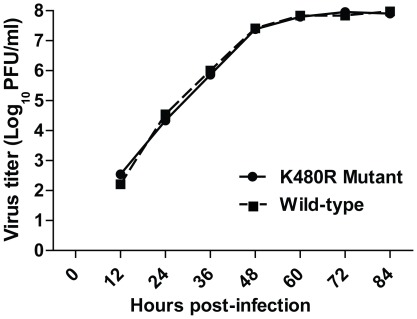
Growth characteristics of wild-type virus and virus possessing the PB1 K480R mutation. Wild-type A/California/04/09 virus and recombinant virus possessing the K480R mutation were used for infection of human Calu-3 cells (MOI = 0.001). Supernatants were collected at specified time points following infection, and virus titers were quantified by plaque assays in MDCK cells. Results shown are averages of three plaque assays.

To further assess the significance of the K480R mutation for virus replication, we generated wild-type CA04 virus and a mutant encoding PB1-K480R. Human Calu-3 cells were infected with a multiplicity of infection of 0.001 of wild-type or mutant virus, and virus titers in the cell culture supernatant were measured at several time points after infection. Both viruses replicated in Calu-3 cells with similar kinetics (**Fig. 5**), and no difference in plaque characteristics was observed. Sequence analysis of the PB1 gene isolated from mutant viruses after replication in Calu-3 cells indicated that the K480R mutation was maintained in the viral population. Therefore, despite slightly enhanced polymerase activity in the minireplicon assays, the K480R mutation did not significantly affect the replication of a pandemic (H1N1) 2009 virus in human airway epithelial cells.

## Discussion

Based on analysis of currently available sequences in influenza databases, influenza viruses with non-consensus amino acids in the four conserved motifs of PB1 protein exist in nature, although rare in number. To assess the replicative ability of these PB1 proteins, we evaluated their transcription/replication in minireplicon assays and found that most of these PB1 mutations abolished polymerase functionality (**Table 3** and **Fig. 1A–D**). Our data support the conclusions by Biswas and Nayak [10] that the four conserved PB1 motifs are critical for replication and transcription. More importantly, our results raise the question of how viruses with these mutations exist in nature. The PB1 mutants tested exhibited similar polymerase activities in both the human and avian cells, arguing against host-specific effects. Therefore, a possible explanation for the existence of natural isolates with PB1 mutations that abrogate polymerase activity in our test system is the presence of compensatory mutations acquired by the respective viruses. These compensatory mutations may localize to the PB1 protein, and/or to other components of the viral replication complex (i.e., PB2, PA, or NP). Additionally, some of the PB1 mutants tested here were found in viruses not closely related to the model strain used (A/bar-headed goose/Qinghai/1/2005; H5N1; BHG). Hence, the respective mutations may not be active in the genetic background of BHG, but function in their authentic backgrounds. Additional experiments would be needed to address this issue. Furthermore, mixed populations of viruses may have been sampled in which a virus with a mutant, nonfunctional PB1 protein was ‘rescued’ by a coinfecting virus with a functional PB1 gene/protein. Finally, we cannot rule out the possibility that erroneous sequences were submitted to influenza sequence databases.

Interestingly, mutation K480R in motif IV of the PB1 subunit consistently demonstrated slightly increased polymerase activity in H5N1 and H1N1 polymerase complexes relative to wild-type (**Table 3**, and **Figs. 1A–D** **,** **2** **and** **4**). The amino acid alteration at this position is considered conservative since lysine and arginine are basic amino acids that share similar chemical structures. As this mutation has been found in several early pandemic (H1N1) 2009 virus isolates, we used reverse genetics to introduce the K480R mutation into the PB1 subunit of the A/California/04/09 (H1N1; CA04) virus strain to assess viral growth characteristics. Although the K480R mutation yielded increased amounts of replication products (**Fig. 2**) and slightly increased replicative ability in the minireplicon assays (**Figs. 1** **and** **4**), the mutation did not affect virus titers in cell culture (**Fig. 5**). We have considered two likely possibilities to explain the discrepancy in replication efficiency results between the *in vitro* assays and growth curve analysis. While the K480R mutation reproducibly enhanced transcription/replication in two different polymerase backgrounds and in two different cell lines, the observed increase (see **Table 3** and **Figs. 1A–D** **,** **2** **and** **4**) may not be sufficient to translate into a detectable rise in virus titer of the CA04 strain. Alternatively, the mutation in PB1 may increase transcription/replication, but concomitantly interferes with another step in the viral life cycle.

Structural data of the PB1 protein, along with the rest of the heterotrimeric polymerase, may help in the interpretation of mutational analysis of these four conserved motifs. To date, there are no crystal structures available of the central region of the PB1 subunit in which the consensus motifs are found [23], [24]. However, based on secondary structure predictions, the ‘invariant’ amino acids are frequently located within or proximal to turn structures that may be crucial for their proper positioning in processes such as cation binding and template specificity [11]. Considering the ‘invariant’ amino acids are embedded in blocks of conserved residues, it is conceivable that the four motifs work cooperatively to form a well-defined functional unit constituting the polymerase module [10], [11]. Therefore, intermotif distances may also be critical for polymerase functionality by providing the correct folding and three-dimensional structure of the protein. Once detailed structural information of the PB1 subunit becomes available, structure-function analysis may help to further elucidate features of primary sequence or potential secondary structures of the four conserved PB1 motifs that contribute to influenza polymerase activity.

Despite lack of functional information on the individual conserved PB1 motifs of the influenza virus, sequence comparison and structural analysis of other RNA and DNA dependent viruses suggest motif III as the core of the transcriptase-replicase activity [10]. This motif is defined by a centrally located SDD consensus sequence for influenza viruses (versus GDD in most other viral polymerases). Common among all RNA-dependent RNA polymerases, the two ‘invariant’ aspartic acid residues are flanked by hydrophobic residues and located in the turn of a beta-turn-beta supersecondary structure [10], [11], [12], [13]. Consequently, motif III has been implicated in several functions including metal and phosphate binding, template recognition, and other catalytic functions. Our minireplicon assay results support the concept of motif III as a key functional domain, as any mutation altering the SDD sequence rendered the polymerase nonfunctional (**Table 3** and **Fig. 1A–D**). We found that mutations S444P and D445G rendered the protein unstable (**Fig. 3**), suggesting a structural role for the amino acids at these positions. However, protein stability (but not functionality) was maintained by serine or cysteine residues at position 445, possibly in combination with additional mutations nearby. Collectively, these findings indicate that the highly conserved SDD motif is critical for protein stability and functionality.

Our analysis of publicly available PB1 sequences suggests that mutations in PB1 motifs I – IV occur more often in avian virus PB1 proteins than in human or swine virus PB1 sequences (with the exception of the K480R mutation which is frequently found in swine virus PB1 proteins). Among avian virus PB1 proteins, more mutations are found in the PB1 proteins of H5N1 and H9N2 viruses than in those of other subtypes (**Table 2**). Viruses of the H5N1 and H9N2 subtypes circulate in poultry, in which influenza viruses are known to mutate at higher rates than in aquatic birds [25]. Thus, the relatively high rate of H5N1 and H9N2 viruses with mutations in PB1 motifs I – IV may reflect the evolution of these viruses in poultry; alternatively, mutations in the PB1 motifs I – IV may render the polymerase complex more error-prone.

In summary, we have carried out a comprehensive analysis of non-consensus amino acids in the four conserved motifs of PB1. Although the mutations tested were reported in natural isolates, most of them abolished polymerase activity, possibly suggesting mixed virus populations and/or the presence of compensatory mutations in the respective isolates. In addition, our study provides more information on the PB1 amino acids critical for catalytic function of the influenza polymerase complex. Such conserved residues comprising the polymerase module may serve as potential targets for anti-influenza drugs that can attenuate infection by inhibiting influenza polymerase activity and thereby, virus replication.
